# Experiences of midwives on pharmacological and non-pharmacological labour pain management in Ghana

**DOI:** 10.1186/s12978-017-0398-y

**Published:** 2017-10-16

**Authors:** Lydia Aziato, Abigail A. Kyei, Godsway Deku

**Affiliations:** 1 Department of Adult Health, School of Nursing, University of Ghana, Legon, Accra, Ghana; 2 Department of Nursing, Pentecost University College, Accra, Ghana; 3 School of Nursing, College of Health Sciences, University of Ghana, P.O. Box LG 43, Legon, Accra, Ghana

**Keywords:** Ghana, Labour pain, Labour, Midwives, Pain management, Qualitative research

## Abstract

**Background:**

Due to the debilitating effects of severe labour pains, labour pain management continues to be an important subject that requires much attention. Thus, this study sought to gain a detailed insight into the experiences of midwives on pharmacological and non-pharmacological labour pain management strategies in a resource limited clinical context.

**Methods:**

A descriptive exploratory qualitative design was adopted for this study which allowed in-depth follow-up of the midwives’ comments resulting in a full understanding of emerging findings. Face-to-face individual interviews were conducted, transcribed and data were analysed using content analysis procedures. Verbatim quotes were used to support the findings.

**Results:**

Midwives employed different pain control measures including pharmacological and non-pharmacological methods such as psychological care, sacral massage and deep breathing exercises. Doctors prescribed analgesics most of the time while in some cases, the midwives independently administered the drugs. They assisted women who had epidural anaesthesia given by anaesthetists. The midwives did not administer adequate analgesics because of fear of side effects of analgesics. Although the midwives exhibited knowledge on drugs used for labour pain management, they did not regularly administer analgesics and non-pharmacological care provided were inadequate due to increased workload. Some of the midwives showed empathy towards women and supported the women. Most of the midwives perceived labour pain as normal and encouraged women to bear pain.

**Conclusion:**

Midwives require regular education on labour pain management and they should pay attention to women in labour individually and administer the care that meets their need.

## Plain English summary

Severe pain during childbirth is a common phenomenon. Thus, we sought to gain an understanding of how midwives in Ghana employ pain relieving drugs and other non-drug measures to relief pain during childbirth.

We used a research approach where face-to-face individual interviews were conducted and these were written and analysed based on the content of the text. The participants’ own words were used to support the findings.

We found that midwives employed different pain control measures including the use of pain relieving drugs and non-drug methods such as psychological care, massaging the lower back and deep breathing exercises. Also, doctors prescribed pain killers most of the time while in some cases, the midwives gave the drugs on their own accord. The midwives helped the specialist to give drugs to provide total pain relief.

The midwives did not give adequate pain killers because of fear of side effects and increased workload although they had knowledge on drugs used for managing pain during childbirth. Some of the midwives thought the pain was normal and encouraged women to bear pain which further contributed to the poor labour pain management. However, some midwives showed empathy to the situation women in labour go through.

We concluded that midwives require regular education on labour pain management and they should pay attention to women in labour individually and give the care that meets their need.

## Background

Midwives play a vital role in the provision of obstetric services to women and babies globally [1]. Midwives require expertise in various aspects of obstetric care such as pain management to achieve safe motherhood [2]. In Ghana, The Nursing and Midwifery Council of Ghana (N&MC) is the body that ensures standards of training and practice of nurses and midwives in Ghana. Training of midwives is done in nursing and midwifery schools across the country. This includes certificate midwifery programmes which lasts for two years, diploma in midwifery which is a three year programme and Bachelor of Science in midwifery which is a four year programme. It is noted that about 57% of childbirths in Ghana are delivered with the assistance of midwives/nurses [3]. In this light, the experiences and effective interventions of midwives on labour pain management is important to explore since inadequate labour pain management continues to be reported [4]. It is noted that within the Ghanaian clinical context, all midwives are responsible for managing labour pain. However, in hospitals where obstetricians are available, midwives administer analgesics as prescribed.

Although pain during childbirth is viewed as a normal process without any threats to a woman’s life [5], much of these experiences rely on the support of midwives during labour pain [6]. The individual qualities of midwives, including the years of professional experiences as well as the number of deliveries conducted may influence labour pain assessment and the methodology employed in managing labour pain [7, 8] However, it is believed that the estimation of labour pain is lower when a midwife has many years of work experience. In the case where a midwife had a lot of personal childbirth experience, her estimation of labour pain is higher [8]. Thus, midwives must always remember pain as an individual phenomenon and employ multidimensional assessment methods for labour pain [9] and manage each woman as an individual [6].

Midwives use analgesics such as opioids [10], non-opioids [11] and epidural anaesthesia [12]. Pethidine is the commonly prescribed opioid analgesic in Ghana [13] because it is cheap, well-known, easy to administer [14]. Nevertheless, there are reports of negative impact of opioids on mother and baby [15] such as nausea, vomiting, sedation [10, 16] and compromised newborn’s respiratory function [17]. Thus, other drugs such as Promethazine is administered to resolve emesis with opioids during labour [18]. Opioid use may be appropriate for older women whose cervix dilates to 3 cm [19]. However, midwives observed that women who are often not given analgesia have short duration of labour [19].

Epidural analgesia induce absolute labour pain relief [17] but it may prolong labour [16]. It is necessary to make available regional analgesia in case women ask for it [20]. Other analgesia used in labour pain management include Hyoscine Butyl Bromide *(Buscopan)* which dilates the cervix [21] and shortens the duration of first stage of labour [22]; Lidocaine is given before an episiotomy and before suturing [23] and Ketamine [24] and Tramadol are sometimes given [25].

The widely used non-pharmacological approaches are deep breathing exercises and positioning [12] perhaps because there is comfort and diversion of attention from the pain. Other methods such as massage decrease labour pain [26], lessen dependence on analgesics [27] and decrease anxiety especially when performed with a comforting touch [28]. Midwives can also give support through praises [29], therapeutic touch [30], as well as explaining or informing women of labour progress [29, 31]. It is noted that insufficient midwives’ support during labour could result in negative childbirth experiences for women [32]. Certain factors such as shortage of health staff, inadequate resources and stressful work setting cause midwives’ burnout and hinder adequate labour pain management [33]. The shortage of health professionals increases workload and subsequently reduces the time staff allocates for pain management [34]. In spite of all these challenges, education of midwives can improve management of labour pain [35].

In Ghana, previous researches in this area were limited and did not deal specifically with midwives experiences on labour pain management, but centered on the childbirth experiences of Ghanaian women [36] and religious practices during labour [37, 38]. Therefore the objectives of the study were to gain in-depth insight into midwives’ experiences on pharmacological and non-pharmacological labour pain management methods adopted in a Ghanaian care setting.

## Methods

### Design

The study adopted descriptive exploratory design to investigate the experiences of midwives regarding labour pain management. Exploratory qualitative approach allows the researcher to explore a phenomenon in-depth.

### Study setting

This study was conducted at the labour wards and maternity wards of a tertiary health facility in Accra, Ghana (Korle-bu. Teaching Hospital –KBTH). The hospital has 2 labour wards and 20 midwives working in these wards were included. All the midwives had a minimum of 3 years’ experience caring for women in labour.

### Target population and sampling

Midwives who had cared for women in labour were targeted and those who agreed to participate in the study were recruited. Purposive sampling was used to recruit midwives who had a minimum of 3 years’ experience.

### Data collection procedures

After gaining institutional access, the first author who is an experienced qualitative researcher conducted individual face-to-face interviews in English within the period of March and July, 2015. Open ended questions were asked and participants freely shared their pain management experiences. The interview guide was developed by the researchers based on the objectives of the study and probes were used based on the responses of the midwives. The interviews were recorded and later transcribed. Participants gave consent for the interviews to be recorded. Field notes were kept and reflexive notes ensured that the participants’ views were faithfully represented.

### Data management and analysis

Data was analysed concurrently during the study using techniques of content analysis which is an inductive approach. The researchers read the transcripts several times which helped them gain full understanding of the participants’ world. The data was coded and similar codes were grouped. Themes and sub-themes were generated [39] and these were used as an organizing framework in NVivo. The data was then exported into the software and this was used to manage the data. The research team discussed the codes, themes and sub-themes and consensus was reached after a review of the raw data.

### Rigour

The rigour of the study was ensured through iterative questioning to follow-up on earlier comments that were not clear. Concurrent analysis allowed a follow-up on emerging findings and this ensured data saturation where no new findings were identified. Detailed field notes and reflexive notes ensured that the views of the research team were not used in the presentation of findings. Direct words were used to present findings and this gave voice to the midwives.

### Ethical considerations

Ethical clearance was obtained from the institutional ethical review board of the Noguchi Memorial Institute of Medical Research of the University of Ghana. The tertiary facility approved the conduct of the study at the maternity unit. All the midwives gave individual informed consent and they signed the consent form. Identification codes were used to represent the midwives such as MW1, MW2 etc. according to their chronologic entry into the study.

## Results

### Demographic characteristics

The participants were 20 midwives and their ages were between 25 and 59 years. The participants had worked for a period ranging from 3 years to 25 years. Their ranks ranged from Staff Midwife to Principal Midwifery Officer. The number of participants who had children were 18 and 2 of them had no children. Majority (17) of the midwives were married, 2 were single and 1 was divorced. Also, 19 of them were Christians and 1 was a Muslim. The midwives were all Ghanaians from various ethnic backgrounds.

### Pharmacologic labour pain management

This theme describes different pain control modalities the midwives employed in the management of labour pain. The sub-themes included: use of opioids and non-opioids pharmacological agents, variations of prescription of analgesics, fear of side effects of analgesics and experiences with local and epidural anaesthesia.

### Use of opioid and non-opioid pharmacological agents

Both opioids and non-opioids pharmacological agents such as Pethidine, Phenergan, Diclofenac, Buscopan, Midazolam, Ketamine, Xylocaine 1% and Tramadol were used by midwives to manage labour pain. Most midwives used Pethidine and Phenergan to sedate or calm the women in labour pain.

*“We use Pethidine 100mg and Phenergan 25mg to calm them so that they can sleep a little …they feel sleepy and the pain at the same time”* (MW1).

Some participants administered Phenergan to prevent vomiting associated with Pethidine. *“…some people do vomit when you give the Pethidine without the Phenergan. So, to avoid the vomiting, we give Phenergan”* (MW10). The midwives sedated the women when cervical dilation was from 0 to 4 cm or before 6 cm.

Diclofenac and Tramadol were also administered to manage labour or incisional pain on account of an episiotomy. They thought that Tramadol had a short-acting effect. *“We give Diclofenac after episiotomy”* (MW13); *“We give Tramadol but it only works for a short period”* (MW20). Others administered Buscopan in cases of ‘*too much*’ or exaggerated labour pain and to help relax the woman especially in cases of cervical oedema.

“*Mostly we give Buscopan to those who exaggerate labour pain”* (MW22); *“… if pain is too much and the cervix becomes oedematous, we sometimes give Buscopan to relax the system”* (MW20).

Midwives perception of exaggerated pain could interfere with effective pain management and individualized pain management.

Other sedatives such as Midazolam and Ketamine were sometimes given but the midwives realized that they were not effective for women who were alcoholics. *“There was this lady that we gave Midazolam and Ketamine to, but they didn’t work because of her alcoholism history”* (MW10). This could mean a woman in labour with a history of alcoholism may experience inadequate labour pain management.

### Variations of prescription of analgesics

The pharmacological agents were mostly prescribed by doctors, but in instances where the doctors were not available, the midwives gave pain killers during labour pain based on their experience and severity of pain expressed by the woman in labour.

“…*we give Pethidine depending on the doctor’s prescription”* (MW10); *“…sometimes when the doctors are not available, depending on how experienced you are and how severe the pain is, we give the painkiller using our discretion”* (MW9).

It was reported that midwives at the District level gave drugs like pethidine independently but in Teaching hospitals, doctor’s approval was required. “*At the District level, I will give the Pethidine because I will not be questioned but because this is a teaching hospital, I will seek approval from the doctors”* (MW2). Most midwives administered painkillers when labour pain exceeded the women’s pain threshold. “*… if the pain is so severe and they cannot bear, …I just give the painkiller”* (MW10).

However, sometimes analgesics were not available for midwives to use during labour and in such cases, the midwives only offered reassurance.

“*Sometimes the ward does not have suppository Diclofenac or Paracetamol to give to the women. If the patients have we will give, so we just reassure them when they are in pain*” (MW2).

This finding indicate that even when prescriptions are given, the midwives may not administer the analgesic because it is not available.

### Fear of side effects of analgesics

The midwives perceived that some women felt dizzy when they administered Pethidine.

“*Sometimes we give* [Pethidine] *if the dilation is from 0 to 4cm”* (MW1); “*Most of the time before the person gets to 6cm we sedate with Pethidine”* (MW10).

*“…some women feel dizzy when Pethidine is given so most of the time we don’t give”* (MW6).

The feeling of dizziness in some women could lead to fear in the midwives and this could interfere with effective labour pain management.

Hence, the sedatives were not given at the late stage of labour when the cervix is fully dilated because of fear of the baby’s respiration system. “*If the labour is eminent and you the midwife you sedate or you give some drugs it will interfere with the baby’s respiration, so we don’t give for fear that the baby can die”* (MW12).

### Experiences with local and epidural anaesthesia

Some midwives used 1% Xylocaine to infiltrate the perineum before an episiotomy was performed. “… *sometimes I give Xylocaine 1% to infiltrate the perineum before I give the episiotomy to aid the delivery and so the woman will not feel pain but sometimes it is not given”* (MW15).

Some midwives perceived that epidural anaesthesia was the best pain relief for labour pain. *“… epidural is the best form of pain relief in labour… the person wouldn’t be shouting or anything”* (MW12). Epidural anaesthesia was not a routine anaesthetic given to all women in labour but it was planned with obstetricians or anaesthetists from the antenatal clinic upon patient request. *“They do not give epidural as a routine; unless the patient talks about it with the obstetrician and they arrange with the anaesthetist”* (MW2).

In some instances, the anaesthetist gave or topped up epidural anaesthesia after the midwives’ assessment confirmed onset of labour by cervical dilatation.

“*Some of the anaesthetists will let us examine the cervical dilatation before they give the top up anaesthetic agent*” (MW3).

The participants indicated that the epidural anaesthesia was topped up when cervical dilatation was less than 8 cm so that the women can have a *bit of pain* to enhance pushing at 10 cm.

“*…when you want to give till 10cm, the person will not push …they give till 6cm, 7cm, 8cm so they can feel the pain to push with it”* (MW10).

Some midwives preferred caesarian section to epidural anaesthesia because it prolonged the second stage of labour leading to resuscitation of the baby or vacuum extraction.

*“Personally …I will go for elective caesarian section than epidural; you have to do resuscitation of the baby most of the time because the second stage is prolonged”* (MW6); *“…and vacuum extraction is sometimes done”* (MW2).

The midwives monitored foetal heart when epidural anaesthesia was administered and it did not usually cause foetal distress.

“*With epidural you just need to monitor the foetal heart well but the drugs used in epidural doesn’t in any way cause foetal distress… I have conducted a few deliveries on women with epidural and the heart rate of the babies were fine”* (MW12).

Epidural anaesthesia was perceived to be costly and mostly preferred by health staff and the affluent in the society during labour*.*

“*…mostly the staffs and those women of high society are given the epidural because it is expensive”* (MW4).

Other participants revealed that some women were left alone after the epidural anaesthesia because of the absence of pain. “*When Epidural is given, they normally leave the women alone because the person is not in pain”* (MW8). A participant who was concerned about the high cost and effect of epidural anaesthesia on the baby suggested further research on Epidural anaesthesia.

“*So if they can research into it, maybe there are some drugs that will work and nothing will happen to the baby as well then they can give those injections*” (MW4).

### Non-pharmacological methods

Some non-pharmacological managements of labour pain were described. Sub-themes identified were psychological care, massage and deep breathing exercises.

### Psychological care

The midwives reassured the women during labour pain to be strong because ‘*the Lord was in control’* and it would be well.

“*I tell them ‘with the help of God, it will be alright and everything will be successful.”* (MW3); *“I tell her to be strong and she will be a bit calmer”* (MW2)

The midwives believed that reassuring and talking to the women to assume side lying positions relieved labour pain. “…at times even talking to the woman reduces pain. You encourage her to lie on her left side… and then you will be with her at short intervals helps” (MW13). Other midwives explained the nature and rationale of labour pain and this prepared their minds for the pain especially the nullipara.

*“I explain to her why she is feeling the pain” (MW4); “We tell them to be patient and the pain will be stronger when the baby is about to be delivered”* (MW6).

“*Those who haven’t delivered before, we reassure them that ‘that is how it is’*” (MW11).

News about the baby’s hairs and head during labour motivated some women who were reluctant to push.

“*We encourage the woman saying “that is your baby’s hairs’ there touch it and see” … then she will say. “Really! The head is there” then she will touch with her hand… then the woman will push”* (MW1).

Some midwives encouraged the women to commit themselves to the Lord or pray for a successful delivery.

“*When they are in pain we tell them oh, give yourself to the Lord. Just pray and the good Lord will help you deliver successfully”* (MW7).

Some women held the hands of health professionals and laughed to a funny comment and this relieved labour pain to some extent.

“*The women sometimes just hold one of the doctors and if you say something funny she will laugh over it and that diverts their attention from the pain”* (MW4).

### Sacral massage

Some midwives massaged the sacral region of the women to relieve them of labour pain. “*When they get the contractions, I …give a sacral massage”* (MW7). In instances where the midwives were overwhelmed by many patients, they taught the women to rub the back to relieve their labour pain, “*I also tell them to rub their back if they can. Usually, they are many and you can’t sit around one patient all day to do sacral massage*” (MW12).

### Deep breathing

Some midwives admitted that during labour pain, they encouraged the women to breathe in and out. “*We encourage them to breathe in and out”* (MW11). They were of the view that breathing through the mouth would supply more oxygen to the foetus but some women did not comply.

“*When you breathe through your mouth, the child also gets oxygen”* (MW13); “*…we tell them to breathe through their mouth. Some of them comply but others don’t*” (MW2).

The midwives advised the women to avoid screaming in order to preserve the energy needed for pushing. “*… We tell them not to make too much noise or else they will lose a lot of energy and not be able to push*” (MW1).

However, the midwives were of the view that they could not manage labour pain effectively because of increased workload.

“*The high workload is not allowing us to manage the pain”* (MW1).

“*I delivered four patients within a short time;…the ward was so busy, that day we were only three nurses and three midwives…it was very pathetic.; …to deliver three others successively. …when you are assigned three beds, you end up taking care of six or eight women”* (MW15).

## Discussion

The current study explored midwives’ experiences on labour pain management using pharmacological and non-pharmacological approaches and these are discussed in relation to the wider literature. Midwives sometimes used a number of analgesics to manage labour pain including opioids such as Pethidine. Pethidine had a calming, sedating effect and is also associated with dizziness and vomiting as confirmed in previous studies [10, 16]. Intense labour pain causes some women to demand Pethidine (Meperidine) and this might have accounted for the high Pethidine use in Obstetrics and Gynaecology units [13, 14]. Use of Phenergan (Promethazine) to counteract vomiting associated with pethidine is congruent with other studies [18]. Midwives sedated women who had 0 to 6 cm cervical dilatation so that they will be able to bear down and the baby will not suffer respiratory depression [17]. This suggests that drugs used during labour could affect both mother and baby which is important for midwives to acknowledge. [35].

Also, the notion that epidural anaesthesia was the best approach for complete labour pain relief is supported [17] but because it was expensive only few women could afford it. Within the context of the study, epidural anaesthesia is not a regular approach to pain management during labour. The authors suggest that epidural anaesthesia should be subsidized so that all women could make a choice for pain relief during labour. The tendency of epidural anaesthesia to prolong labour [16] in the second stage, leading to resuscitation could be the reason why some midwives indicated that they preferred caesarian section. In this case, midwives may be quick to resort to other pain relief measures and suggest incisional procedures such as episiotomy and caesarean section. Those who reported that the absence of pain after giving epidural warranted no further monitoring may not identify any foetal distress with epidural anaesthesia.

It was realized that 1% Xylocaine was sometimes used to infiltrate the perineum before an episiotomy and suturing as supported by previous studies where Lidocaine was used [23]. Also, Tramadol and Diclofenac were used for incisional pain but it had a short-acting effect and maternal side effects of tramadol has been reported [25]. Hyoscine butyl bromide (Buscopan) dilates the cervix [21] and shortens the duration of the first stage of labour [22]. Midwives administered Buscopan when labour pain was severe. Women who experienced no effect with the analgesics were believed to engage in alcoholism or smoking as reported in other medical conditions [40, 41].

Doctors mostly prescribed analgesics but in instances where the doctors were beyond reach, the midwives gave pain killers as reported in other studies [14]. Midwives at the District level gave drugs like Pethidine independently but in Teaching hospitals, the doctor’s approval was almost always required. This could be due to the fears of the negative impact of these drugs on labour and the baby [15]. In some contexts, Pethidine can even be administered by a midwife at a health center, Level B2 [42], hence freedom to administer certain drugs is dependent on the context.

Non-pharmacological approaches used included reassurance and demonstration of religiosity as reported in previous studies [38]. Some midwives showed empathy and administered painkillers when pain was very severe. This feeling of empathy shapes the midwives’ approaches to labour pain assessment [8]. Labour pain management is influenced by the midwives’ professional experiences regarding number of deliveries conducted [7, 8]. A midwife who has gone through labour pain is more likely to demonstrate higher estimation of labour pain [8] and could have empathy for labouring women [8, 43, 44].

Midwives also encouraged women to assume the side-lying position to relieve pain. Others massaged the sacral region to relieve them of painful contractions of labour and they taught them as well to do it themselves. Similar findings revealed that massage decreases labour pain intensity [26] and helps to lessen dependence on pain reliefs [27]. Some midwives explained and shared with the nullipara the nature and rationale of labour pain in order to prepare their minds for the pain. It was realized that the midwives encouraged the women to breathe in and out through their mouth during painful contractions in order to supply more oxygen to the foetus as supported by previous researchers [12]. Breathing through the mouth prevents maternal exhaustion beside supplying the foetus with oxygen. Therefore, midwives should highlight deep breathing exercises in their care of women in labour.

Inadequate labour pain management was attributed to heavy work load which prevented the staff from managing pain as supported by Nyamtema [34]. Hildingsson [33] reported that shortage of health staff, inadequate resources and stressing work settings cause midwives’ burnout. Shortage of health staff such as midwives presents an array of problems that stakeholders and policy makers should strive to address. The government and the Ministry of Health of Ghana should ensure adequate staff on labour wards to provide an enabling environment for the midwives to work effectively [3]. Perhaps the heavy workload and lack of resources contribute to inadequate knowledge on labour pain management. The findings in this study confirms the need for vigorous efforts by all stakeholders to assist midwives to manage labour pain effectively at all levels of the health care delivery system.

### Strengths and limitations

A strength of the study is that, the method employed enabled researchers to obtain in-depth information from participants by using probes to follow up on findings identified. However, this study did not employ clinical observation of the midwives to triangulate their experiences. The results of the study may also be limited as the study was conducted in a resource limited setting, therefore, the results cannot be used to a high resource setting. Future research should take into consideration the limitations of this study and involve other health professionals who manage labour pain. The findings may be useful to develop interventions that can improve labour pain management and also help in developing appropriate policies for labour pain management. Future research should compare labour pain management at different levels of the health care delivery system including private and government own facilities. This will provide evidence to institute context appropriate interventions for labour pain management.

## Conclusion

The findings provide evidence for all midwives to be circumspect and monitor foetal heart rate at regular intervals to forestall any avoidable complications. The pharmacodynamics of different drugs used in labour call for continued education and monitoring of effects of drugs on women. The findings further draw attention to the need for midwives to be empowered to educate pregnant women on labour pain. Adequate staffing and access to analgesics should be ensured to enhance labour pain management efforts of midwives. Similar research is needed in high resource settings as the study was conducted in a limited resource setting.

## Abbreviations

KBTH: Korle-Bu Teaching Hospital
